# Let’s play! Gamification as a marketing tool to deliver a digital luxury experience

**DOI:** 10.1007/s10660-021-09529-1

**Published:** 2022-01-17

**Authors:** Matilde Milanesi, Simone Guercini, Andrea Runfola

**Affiliations:** 1 Department of Economics and Management, University of Florence, Via delle Pandette, 9, 50127 Florence, Italy; 2 Department of Economics, University of Perugia, Via Pascoli, 20, 06123 Perugia, Italy

**Keywords:** Luxury, Gamification, Digital experience, E-commerce, Retail

## Abstract

This paper aims to investigate the features of gamification as a digital marketing tool to deliver a digital luxury experience. The paper employs the qualitative methodology of case study and presents a case of a multi-brand luxury company adopting gamification through the development of a game app. Four features of the gaming luxury experience are discussed: individual and collective gaming experiences, exclusiveness through rarity in luxury gaming, social networking and virtual influencing marketing mechanisms, and cross-fertilization between gaming and e-commerce. The paper expands the knowledge on gamification by highlighting its main features as a digital marketing tool for luxury companies and the benefits it can bring in terms of consumer experience, engagement, and sales. It also contributes to studies on luxury companies by discussing gamification as a means to create digital luxury experiences, particularly suitable for the new generations of consumers, such as Generation Z.

## Introduction

The COVID-19 crisis has heavily affected the luxury goods industry in 2020. The findings from the 19th edition of the Bain & Company Luxury Study, released in collaboration with Fondazione Altagamma, the Italian luxury goods manufacturers' industry foundation, show that the personal luxury goods market experienced a large drop in 2020, with a loss in sales of 23% compared to the previous year. The overall luxury market contracted at a similar pace, and it is expected to recover by 2022–2023 [1]. The turmoil of COVID-19 has been the trigger for change for the luxury industry, both for companies and consumers. For luxury companies, the COVID-19 crisis accelerated the online sales that nearly doubled from 12% in 2019 to 23% in 2020, and the online is expected to become the leading channel for luxury purchases by 2025, especially for younger generations (Millennials and Generation Z) [1]. Thus, a suggestion is to put digital, besides internationalization [2], at the center of a company's business model. In the case of luxury, since luxury consumers are used to high standards in-store services, a personalized and engaging digital experience should be created [3].

These last months have led to identifying new ways to engage consumers for many luxury fashion brands. In a year, 2020, made up of digital catwalks, live shows on social networks, and new e-commerce mechanisms, such as Shop Streaming, a phenomenon that has undergone a significant acceleration in luxury fashion is gamification that consists of turning customers' everyday interactions into games for business purposes [4]. Gamification makes it possible to provide an immersive experience by developing innovative and customized customer journeys and high levels of engagement [5, 6]. However, most research investigates the general effects of gamification on branding e.g. [7–11] but there is a lack of studies that consider gamification as a strategic digital marketing tool and investigate the features to deliver digital experiences. Additionally, while conceptual papers have been recently developed e.g., [12, 13], this issue requires additional empirical evidence to better understand the implications of the adoption of gamification for companies.

In the luxury fashion domain, numerous *Maisons* have chosen gamification as a tool to express their values interactively, especially with younger generations. According to the data published by Fashionnetwork,^1^ people between the ages of 18 and 34 will represent 50% of the luxury market in 2025, up from 32% today. Therefore, it is a target on which fashion luxury companies should invest through new and original tools to deliver a luxury digital experience. During the Paris Fashion Week in 2020, Christian Louboutin created a virtual presentation with Zepeto, an app designed in South Korea with which people can interact on a social network, but with a three-dimensional avatar created from a selfie. In March 2020, Valentino created, together with Nintendo and photographer Kara Chung, an idyllic reality populated by deserted islands on which to build houses and shops, engage in gardening or go virtual shopping. Hermes created the Saut Hermès game, a virtual version of the annual show jumping competition at the Grand Palais in Paris. Burberry has entered the world of video games by including the 'B-Bounce' game on the website's homepage, where it is possible to accumulate points and even win items from the collection. Gucci has launched, within its app, the 'Gucci Arcade' platform. It is a section dedicated to video games in the style of the 70 s and 80 s. Game apps were also created, such as Drest and Covet Fashion. They have entered into agreements with the leading global luxury brands and online luxury fashion multi-brand retailers, such as Farfetch.

Thus, despite the numerous examples of the integration of gamification into luxury companies' marketing strategies, the academic research on gamification and luxury is limited and mainly focused on branding e.g., [7], but there is a dearth of empirical evidence supporting the features and implications of gamification for luxury companies, especially in terms of delivering a digital luxury experience. Additionally, while there are many studies on online shopping, how online experiences are created and managed by companies, especially those in the luxury industry, is an under-investigated issue Homqvist et al. [14]. To fill the abovementioned gaps, this paper aims to investigate gamification by adopting the perspective of a luxury company integrating gamification tools in its marketing strategy. Thus, the paper answers the following research question: *what are the features of gamification as a marketing tool to deliver a digital luxury experience?*

To this aim, the paper employs the qualitative methodology of case study and presents a case of a multi-brand luxury retailer based in Italy. The company has recently launched a game app for international customers, representing a unique case in the current luxury panorama. The paper provides a twofold contribution. First, it expands the knowledge on gamification by highlighting the features that gamification, as a marketing tool, should have in the context of luxury. In doing this, the paper answers the request of providing empirical investigation on gamification aimed at understanding the tangible benefits it can bring to companies in terms of consumer experience, engagement, and, to a later extent, conversions into sales, which are particularly relevant for luxury companies with an e-commerce website. Second, it contributes to studies on digital luxury experiences particularly suitable for the online world and the new generations of consumers, such as Generation Z. The next section of the paper analyzes the marketing content emerging from the literature on gamification to understand what is known so far about the adoption of game design elements in a non-game context such as the marketing one. Section 3 concerns the methodology of the case study. Section 4 presents the case analysis. Finally, the paper discusses findings and concludes with theoretical, managerial implications, and future research directions.

## Theoretical background

### The application of gamification to marketing contexts

Gamification uses game design elements in non-game contexts to make an application, product, or service funnier, motivating and engaging [15]. Zichermann and Linder [4] defined it as *the art and science of turning your customer's everyday interactions into games that serve your business purposes* [4], p. 20). Some scholars describe gamification as several design principles, processes, and systems that can be used to influence, engage and motivate individuals, groups, and communities, drive their behaviors, or generate the desired effect [16, 17]. The dynamics, gaming techniques, and game-style rewards have been transferred from the gaming software origins to business contexts [6], with the initial aim of increasing customer engagement [11]. Thus, gamification implies the development of applications with games features and has become a fast-emerging trend in non-gaming contexts, especially marketing [18].

Most marketing studies on gamification have focused on branding and outlined other benefits of applying gaming mechanisms. Gamification seems to be positively associated not only with brand engagement [19] but also with brand attitude [11], brand awareness [18], brand co-creation experiences [9], and brand love [7]. It has also been shown that the application of game dynamics in marketing contexts allows to achieve greater customer loyalty [20] and to increase sales [21, 22]. Thus, gamification tools can be used on e-commerce websites to create content, generate conversion, and promote loyalty [23].

In addition to the benefits of gamification related to branding, a relevant issue related to gamification concerns game mechanics and game design-related gaming motivations: immersion-related, achievement-related, and social interaction-related dimensions [24–27]. The immersion-related features concern the player's immersion in self-directed, inquisitive activity through game mechanics such as roleplay, avatars, narrative structures, etc. The achievement-related features enhance the players' sense of accomplishment through game mechanics such as challenges, missions, goals, badges, progression metrics, etc. Finally, the social interaction-related features attempt to enable the players' social interaction through game mechanisms such as teams, communities, groups, and competition [25, 28]. In the marketing context, Xi and Hamari [10] show that achievement and social interaction-related gamification features are positively associated with emotional, cognitive, and social brand engagement, with a positive effect also on brand equity. Thus, the authors conclude that gamification appears to be an effective technique for brand management.

Research on gamification suggests that motivational and emotional involvement during playing can reach very high levels [29]. Concerning motivation, a distinction is between intrinsic and extrinsic motivation [30]. Intrinsic motivation concerns internal achievement related to positive feelings and enjoyment, while extrinsic motivation is more related to external rewards like money or other tangibles (Denny 2014). Yang et al. [11] observe that *as gamification marketing process is normally committed to instill products or brands information to users, it is a drive for participants to learn the information and further join or continue an action—in our case, engaging with gamification—because of the effects it has. Therefore, when people are intrinsically motivated, they have a genuine desire for the activity itself and enjoy it tremendously* [11], p. 460). The role of intrinsic motivation is connected to loyalty. In particular, it has been observed that many marketing attempts to engage consumers, including loyalty programs, membership systems, and point-based management, which are based on monetary and tangible awards, are less efficient in generating loyalty in the long term compared to marketing strategies based on intrinsic motivation, such as gamification [31, 32].

### Gamification and digital experiences

With consumers surrounded by new and fast-evolving technologies, more and more selective, and ponder how to spend their time and money, companies are put under pressure to adopt marketing tools capable of attracting consumers' attention and engaging them [11]. As outlined in the previous section, consumers are willing to adopt gamified systems in search of fun, rewards, competition, social interaction, and a sense of membership in a community. Nobre and Ferreira [9] also suggest that gamification can be used as an innovative branding tool to promote consumer participation and interaction in brand experiences.

The creation of experiences is a compelling issue and a relevant factor of success for all companies, especially when such experiences must be translated into a digital context with a proper shopping atmosphere [33, 34]. This aspect is particularly true for luxury companies, for which digitalization and online sales are still a challenge for exclusivity and identity [35]. Batat [36] agrees that gamification is one of the possible digital marketing tools for luxury companies to deliver luxury experiences. Desmichel and Kocher [37] suggest that multi-brand luxury stores should take more significant effort to promote offline and online hedonic shopping experiences to make consumers less price-sensitive and improve profitability. Insley and Nunan [38] observed in their study on online retailers that the transfer of customer experience from an offline to an online context creates challenges, as many of the factors that create a successful physical shopping experience are lost when moved to the online world. However, gamification can make up for this issue by creating gameful experiences [13], as retailers—Amazon and ASOS are two examples—are increasingly applying game techniques in their online store to create reward mechanisms and turn online shopping into an entertainment experience [38]. Mobile technologies can enhance such an experience [12]. Online retailers often apply basic game mechanisms, such as reward mechanisms for completing specific tasks [39]. It appears that the application of gamification in an online retail context is not about gaming, but it concerns the addition of game elements, such as reward mechanisms, to enrich the existing shopping process. A step forward could be the development of brand-related game apps to deliver brand-related online experiences that make use of mobile technologies and apply more complex game mechanisms [40].

## Methodology

To investigate the features of gamification as a marketing tool to deliver a digital luxury experience, case study methodology was selected. Since there is a dearth of empirical studies on this topic, especially in the luxury domain, the case study was deemed the appropriate methodology to gather in-depth data to reveal the features of gamification developed by luxury companies. Hence, the nature of the research question and the novelty of the subject called for the choice of an explorative methodology [41]. The choice of the case study method also relies on the interest in reconstructing the dynamics of a process (the origins of the idea, the design, and of a game app in the case under study), which is an objective for which the case study method appears particularly suitable [42]. The paper proposes a single case study to reconstruct the features of a game app developed by an Italian company—MZQ from here on for confidentiality reasons—to deliver a digital luxury experience to its customers.

The MZQ case was purposely selected for two reasons. First, the company is one of the most relevant Italian multi-brand luxury retailers with a strong international profile; it represents an emblematic case for the development of its game app, called VER5 (a fictitious name), which develops a gaming universe centered on luxury products and closely connected with the company's e-commerce website. Thus, the case has been selected for its revelatory potential.

Data collection started in 2013, in the context of a broader research process initiated before the introduction of the game app, aimed at monitoring a company—MZQ—the presented characteristics of particular interest for the success and rapid growth achieved in the luxury fashion e-commerce, starting from a traditional luxury clothing retail business. The first in-depth semi-structured interviews were initiated by one of the authors in November 2013. They continued over the years, with the involvement of the other authors, according to the scheme indicated in Table 1. The initial interviews concerned the international development of the company, its business model, and its marketing strategies. For this study, such content has been employed to depict the company profile described at the beginning of the next section.

**Table 1:** Data collection

Source	Method	Dates	Data
Entrepreneur and owner	Face-to-face meeting	April 2015 February 2016	About 1 h of colloquium
General director	Face-to-face interviews Email exchange	November 2013 October 2014 November2015 March 2017	About 5 h of interviews Contents of 8 e-mails
Marketing director	Face-to-face and telephone interviews Email exchange Videoconference	February 2014 October 2014 June 2015 November 2017 September 2019 January 2021 February 2021 September 2021	About 9 h of interviews Contents of 9 e-mails
Web designer	Videoconference	February 2021	About 2 h of interviews
Annual reports and internal documents	Content analysis	7 annual reports, from 2013 to 2020 Internal documents from 2019 to 2021	About 360 pages of text and schemes
Events	Participation	May 2015 January 2016	About 6 h of participation
Company website	Content analysis	Different moments from November 2013 to October 2021	View of webpages in 8 different languages Products and promotions
Game app VER5	Active participant observation	From February to October 2021	Download and play, game mechanisms

Starting from 2017, when the company began to think about the development of a game app, the interviews focused on gamification. Interviews involved different informants (entrepreneur, general director, marketing manager, web designer) to ensure multiple perspectives. All interviews, conducted face-to-face, by telephone or videoconference, were recorded and transcribed and supplemented with the exchange of emails. However, a hallmark of rigorous case study research is the use of multiple data sources that enhance data credibility and allow triangulation [43–45], with each data source as one piece of the *puzzle* that contributes to the researcher's understanding of the whole phenomenon (Yin 1994). Thus, in addition to the interviews, data collection included: annual reports, internal documents provided by the company, participation in MZQ events, company website, and other online documents such as articles in the specialized press and published interviews. The participation to the company's events represented a social activity that allowed interactions with the entrepreneur and other managers in a less formal way that, in the following steps of the research, simplified the establishment of channels of communication and the researcher–manager interface [46, 47].

Furthermore, to better understand the functioning mechanisms of the game app, we downloaded it and played it for nine months. This active participation made it possible to fully understand the functioning mechanisms (creation of the avatar, challenges, levels, awards, community interaction). It would not have been possible to grasp such details from the interviews and secondary material only.

Data from the abovementioned sources were the object of systematization by developing a complete chronology of events involving the company, its growth as a luxury multi-brand retailer, and the development of the game app. This analysis has been translated into the company profile and the origins of the idea of VER5. We then proceeded with an in-depth analysis of the process of design and development of the game app. In this phase, data were analyzed through a coding procedure into common themes and pattern matching [48]. We analyzed the material individually to bring out some themes corresponding to the main features of the game app, then discussed them to converge on common themes with full agreement. The structure of the next section is based on the following themes: individual and collective gaming experiences, exclusiveness through rarity in luxury gaming, social networking and virtual influencing marketing mechanisms, cross-fertilization between gaming and e-commerce. Thus, in this paper, the case study emerged from a research process and interaction path with the company, based on two levels: the first level concerns the company within which the process of generation and dissemination of the game app is developed,the second level relates to the game app project, with attention to its features and outcomes. The study of the company represents the context in which the gamification process develops.

## The case of MZQ and VER5

### MZQ and the launch of VER5

MZQ is a luxury multi-brand retailer with one boutique in an Italian city and an e-commerce website. The company's origin dates to the 1920s with the opening of the boutique. In 1999, the company made the pioneering decision to invest in the digital channel through the development of an e-commerce website to sell luxury items, which today generates about 90% of the company's overall turnover. Over time, MZQ has become one of the leading players in international luxury fashion retailing. The e-commerce website is developed in 9 languages and sells in 130 countries. In 2020, it recorded over 100 million hits from all over the world. The total turnover of MZQ stood at around 145 million euros in 2019, with a preponderant weight of sales on foreign markets and a lower incidence of the domestic market. The company's decision to invest in gamification dates to four years ago, when in 2017 MZQ decided to develop a mobile fashion game application through an agreement with a specialized gaming company. MZQ launched VER5 at the end of 2020 after a testing period. The app initially included womenswear in the game, menswear was added a few months later. It is currently available in two languages (Italian and English). As underlined by the company, it was the first app based on an *innovative ecosystem that offers users the opportunity to combine the gaming, social and shopping experiences* (source: company's internal report). As one of the informants points out, *the game app represents an element of absolute novelty in fashion and luxury*. Through their avatar, the app users *take part in the challenges, collect, socialize and buy favorite items by creating virtual wardrobes*, continues the interviewee, who emphasizes that *the intention was not to create a fashion game app exclusively for branding purposes, but a new channel to generate sales in the e-commerce website*.

### Some results of VER5: engagement, retention, and revenues

VER5 has reached some relevant results. They can be considered both in terms of users' engagement in the game app and contribution to turnover. Concerning user engagement, the objectives were to create a community of players and offer a new digital experience to consumers. The company succeeded in creating a community of players. After a few months from the launch, the app reached a community of approximately 50,000 players. While managers estimated to reach 200,000 players at the end of the year, in October 2021 the app already reached 350,000 users. Currently, the primary users of the app are in the age group 18–24 years (40% of the total), followed by users in the age group 25–34 (30% of the total), users in the age group 35–44 (17% of the total), users in the age group 45–54 (10%), and over 55 (3%). Before the extension to the menswear collections, which happened during 2021, one informant pointed out, *although the gaming platform currently includes only the female world of the company retail assortment, about 20% of users are men, which confirms the extreme interest in luxury and gaming also among this target*. After an initial launch phase in the national market, data shows an increasing share of international users (30% of current app users). The key performance indicators (KPIs) show a significant positioning of the app. There is a noteworthy engagement performance with percentage rates of daily active users above 20% and weekly stickiness of more than 50%, which is a high engagement compared to the average rate of free-to-play games (source: company’s internal report).

The one-day retention rate (the retention of players after the first day of gaming) is nearly 32%, which is above the average of free-to-play games (source: company’s internal report). In addition, the average time spent on the app by users is ten times compared to that spent per session on the e-commerce website. The company classifies users into three prevailing segments: those who use the app as a source of inspiration for their fashion style; those who use the app to become popular through the game; those interested in both fashion and gaming that later purchase through the app.

The app represents a means to generate additional sales for the e-commerce website. It also represents a new channel for generating direct revenues. Regarding additional sales in the online store, conversions from the app to the e-commerce website have already generated a turnover exceeding one million euros. It is also interesting to note that, on average, those who are already MZQ customers tend to double their orders since they start playing with the app. Hence, additional sales refer to both new sales from new app users and upselling from existing customers. Furthermore, the app generates direct revenues, as users can have in-app purchases. For example, they can buy virtual currencies to play the game. Users can spend virtual currencies to buy fashion items (even from other players) and regenerate (for reusing) digital items already collected, as the number of uses within the app of any item is limited. The company also aims to create new revenue sources through the app, such as those generated by collaboration with potential partners (fashion brands) or advertising in the game. Four main features enable the company to pursue these results, as discussed in the following sections.

### Individual and collective gaming experiences

The game design developed by the company includes different levels of players' involvement and, consequently, different luxury gaming experiences. Users can jointly go through individual and collective gaming experiences. At the individual level, the app allows players to create customized virtual avatars and outfits. Players can create outfits by collecting virtual replicas of real products from more than 600 designers and fashion brands sold on MZQ e-commerce. As noted by one of the interviewees, *this lets the consumers experience an individual sense of luxury, by personalizing the avatar and expressing personal fashion styles through the selection of the luxury fashion items proposed in the app*. This essential app feature represents the first dimension of emotional engagement. The game then can be experienced individually, with users that can play customizing both the avatar and the outfits. At the collective level, the app proposes missions and periodic challenges where users can compete. During challenges, for example, users can participate and interact, creating ad-hoc outfits. Participating in the challenges contributes to the users' ranking in the app. Some challenges are free-to-play and concern various topics (such as competitions about favorite total-look for a gala dinner). Challenges end with winners selected by users that positively or negatively evaluate the outfits of other players. In this way, the game, from an individual level (personal avatar), becomes collective (avatars that interact and compete in challenges). The gaming experience, therefore, mixes individual and collective experiences.

### Exclusiveness through rarity in luxury gaming

The concept of exclusivity is crucial as a mechanism capable of generating a luxury gaming experience. Exclusivity is achieved in the game through the rarity of the collectible items. This aspect of fundamental importance differentiates the app from other gaming experiences since it allows the company to maintain a luster even in the mobile platform. In other words, exclusivity permeates the gaming experience and feeds the interaction mechanism between users and the app usage. As an interviewee notes, *we implemented different rarity levels of the products in the game*. The game is free-to-play, but the use of virtual currencies makes it possible to buy fashion boxes with rare items. Rare items can let players differentiate their outfits and give bonuses to win challenges. Hence, the way in which exclusivity permeates the game is twofold. First, users can pursue hedonic goals by collecting more rare items as possible to create exclusive outfits. At the same time, exclusivity feeds players' competitive goals by using rare items during challenges. Furthermore, players can use virtual currency to buy (and sell) items through a market-based mechanism. In this sense, the rarity concept evolves through the interactions among players, as they can buy items from other users.

Exclusivity is then a key driver of app usage. The app measures the exclusiveness of players' virtual wardrobe by assigning a *rarity score that shows the percentage of rare items collected by the player compared to all those available in the game app*. In addition, the company releases new fashion items in the game app every week, constantly feeding the players' search for rare products. One of the main strengths is the exclusive fine selections of fashion items of luxury brands that the company proposes in the game app. One interviewee observes that *the fact that MZQ is a multi-brand retailer allows fueling the rarity by entering a broad set of alternatives into the game*.

### Social networking and virtual influencing marketing mechanisms

Social networking and virtual influencing enrich the luxury experience offered by the game app. The interviewees stress the intention to exploit social networking mechanisms to strengthen players' engagement. Beyond the individual gaming experience, MZQ conceived VER5 as a social network. Consequently, it has implemented mechanisms to stimulate the creation of a community of users who interact with each other and discuss fashion issues.

The app includes a social network section. The company feeds this section several times a week with posts about information and updates on the world of fashion or game news. In this way, it stimulates comments and reactions from users. Additionally, in this section of the app users can post their avatar with different outfits to share them with the community. Therefore, in addition to company-generated content, the social section of the app is enriched by user-generated content, thus representing a hybrid social network that combines company posts and user posts.

Hence, there is the possibility of leveraging the community to become fashion influencers through avatars. The outfits created can be judged by other users. In this sense, each user can potentially increase popularity within the community by showing outfits and moodboards of the items collected, meaning that each virtual avatar can have a base of followers and receive reactions and comments from other users. Interestingly, there is a mix between virtuality and reality, namely between a virtual entity (the avatar) and the real person (the user). In essence, as an informant points out, the user can *combine gaming with social networking and virtual influencing* by using the virtual avatar. It is a peculiar aspect that takes its cue from very recent fashion trends, as one informant explains, *think for example that Lil Miquela, a virtual influencer created via computer graphics, reached 3 million followers in three years*.

### Cross-fertilization between gaming and e-commerce

The gaming experience allows players to visit the e-commerce website to make purchases. Hence, the gaming experience integrates luxury shopping experiences. The app offers a close connection with the e-commerce website in all the sections of the game. Users can buy real versions of virtual items collected in their virtual wardrobe. Simply by clicking on the item, they can buy it on the MZQ e-commerce website. Hence, the luxury shopping experience is a fundamental component of the app. As an informant points out, *users spend on average 30 minutes a day to play on VER5, which is about ten times more than the average time spent per session on the MZQ e-commerce website. The longer the user spends time on the app, the more are the conversions to the e-commerce*. Hence, while the company conceived the app as a channel capable of generating autonomous revenues, at the same time it represents a new channel to promote the e-commerce website and generate sales. For example, users can collect points that are part of the loyalty programs of the e-commerce website. However, the connection between the e-commerce and the app is two-way, as the app benefits from the e-commerce website too in terms of promotion and new items for the game.

## Discussion

### Digital luxury experience through gamification: three co-existing levels

**Fig. 1:**
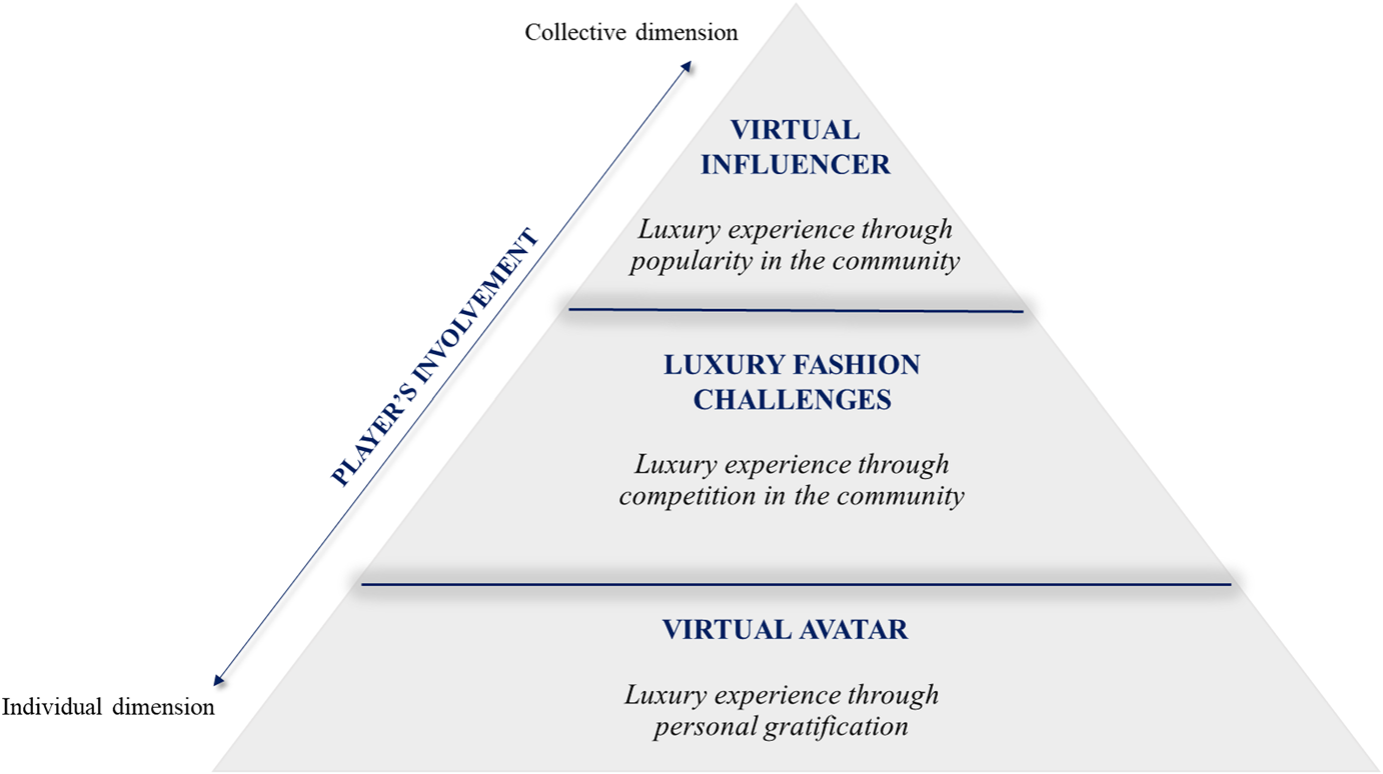
The levels of luxury gaming experience

The MZQ case with the development of the VER5 game app allows us to advance some reflections on the relationship between gamification and the digital luxury experience. Our study shows three levels of players' involvement that bring to a satisfying experience. These levels co-exist as the users can move from one to another while playing. At each level, gamification feeds the digital luxury experience with a different mechanism. Figure 1 shows the three levels and how gamification shapes the digital luxury experience.

At the first level, players can create their virtual avatar to start their immersion in the game. The avatar represents a virtual transposition of oneself and, therefore, allows a greater immersion and involvement in the game from the beginning. Creating a customized avatar is consistent with a playing mechanism that allows pursuing aesthetic and hedonic goals. Players can experience their interpretation of luxury styles by creating virtual outfits and enhance this experience by continuously using new items. Hence, the luxury digital experience is connected to virtual interpretations of oneself and personal gratification.

The second level of involvement refers to the possibility of competing in fashion challenges. Players can test their styles, challenging other players. Thus, gaming is not only an individual experience, but it also implies participation with others while playing. Therefore, this second level contributes to players' engagement through competition, providing interaction and rewards for future challenges. The mechanism that guides competition is the items' rarity. Thus, exclusivity—a typical feature of luxury positioning—represents a pillar of the competition between players. The luxury digital experience is then competing and interacting with others by taking the lever of exclusiveness.

Finally, there is a third level of involvement. Players can aspire to be virtual influencers. They can become fashion trendsetters of a community of players. Playing the game with others lets players' popularity increase through the proposed outfits and styles. As for any social network, users can post their virtual avatars and generate followers in the community. The engagement and the collective dimension in this level reach the top and the luxury digital experience is expressed as the player's ambition to lead a community of followers.

The three levels described are consistent with the literature on gamification that distinguishes three categories of game mechanics and game design-related gaming motivations: immersion-related, achievement-related, and social interaction-related dimensions [24–27]. In the case study, we found all these three dimensions. First, VER5 users can pursue immersion-related motivations by customizing avatars and outfits. Second, they can pursue achievement-related motivation by participating in challenges. Finally, they can reach social interaction-related goals by posting, commenting, and becoming popular influencers in the community of players. In the case of VER5, these three experiential dimensions co-exist, as players can contemporaneously achieve all three dimensions. They are not mutually exclusive, but players can jointly achieve them in the game.

### Designing gamification for a digital luxury experience

The case allows putting forward some considerations about the research question of this study: *what are the features of gamification as a marketing tool to deliver a digital luxury experience?*

From the theoretical point of view, we propose four features that characterize gamification for a satisfying digital luxury experience. We argue that the interplay among these features allows understanding how to design gamification for luxury companies.

First, gamification provides *different levels of digital experience*. Luxury requires innovative experiences [49] and gamification represents a source to deliver new and playful customer experiences. While previous literature [24–27] has pointed out the importance of immersion-related, social interaction-related, and achievement-related features, our study emphasizes the need to treat them not as alternatives but as co-existing dimensions of the game. An innovative digital luxury experience implies the offering of a set of interconnected experiences. In other words, the co-existence of different dimensions drives the luxury gaming experience. Furthermore, considering the case of online multi-brand retailers that operate globally, this gamification feature represents a further dimension of the overall luxury retail strategy [33].

Second, gamification in the luxury context needs *exclusivity as a gaming thread*. The academic debate posits that one of the crucial aspects of luxury is the need to maintain the character of exclusivity, especially for retailers that target global markets and new luxury consumers as Generation Z and Millennials. Therefore, delivering exclusivity online is a real challenge for luxury companies [35, 36]. The case study shows that the app has exclusiveness as its fundamental feature and the central dynamic of the game. The game design implements mechanisms and tools (such as the exchange of rare items among players) that allow consumers to explore and exploit the rarity of virtual items. For players, while exploring exclusivity calls for finding new items and outfits within the international retailer's assortment, exploiting exclusivity implies benefiting from it within the game. Therefore, like in any other offline and online activities of luxury companies, the gaming experience finds expression in the searching for exclusivity.

Third, gamification involves *social networking and virtual influencing*. Previous literature has emphasized the importance of intrinsic motivations related to gamification [11]. We argue that gamification in luxury goes a step forward along the motivation continuum. The academic debate widely accepts the strategic role of social networks and influencer marketing [50, 51]. We argue that luxury gaming can exploit social networking and influencer marketing as additional motivations for consumer engagement and this is essential to target younger consumers. Considering that younger generations show great interest in social networks, social influencing, and mobile games, we argue that the interplay between gamification, social networking, and virtual influencing represents the keystone to reaching these consumer targets globally. Thus, gamification can ensure a connection with consumers who will drive luxury consumption in the future.

Fourth, gamification benefits from *cross-fertilization between gaming and e-commerce*. We argue that gamification represents a new channel to meet sales objectives. While previous literature has showed the role of gamification as a marketing tool for branding purposes [9], this study also considers gamification as a channel to renew and reinforce e-commerce strategies. Therefore, gamification can jointly achieve branding and online sales aims and it constitutes a driver for luxury companies' business model development, especially for online multi-brand retailers, as it allows expanding online sales on a global scale [23]. Gamification is an opportunity for the e-commerce website to benefit from a playful online shopping experience. At the same time, the e-commerce website may feed the game with new exclusive items and new content to support innovativeness and exclusivity. Thus, gaming and shopping experiences co-exist and co-evolve together in the interaction between game apps and e-commerce websites.

## Conclusion, limits, and avenues for future research

The paper allows drawing some managerial implications. Gamification can be an appropriate digital marketing tool for all those companies that intend to involve their consumers and increase their level of engagement. Specifically, gamification can take on different characteristics and levels of complexity, from adding game elements in order to enrich the existing shopping process to developing ad-hoc brand-related game apps aimed at delivering brand-related online experiences through mobile technologies and more complex game mechanisms. Marketing managers must consider the need to develop specific skills in the field of mobile technologies and game design, the latter being so specific that they require partnerships with specialized companies such as game developers. Moreover, gamification as a digital marketing tool should be considered by online multi-brand retailers, especially in the luxury and fashion sectors as, in recent years and after the pandemic crisis, they have to face an increasingly harsh online competition at a global scale. The competition is not only between online multi-brand retailers but also between them and the global luxury brands that increasingly invest in e-commerce websites and marketing tools capable of engaging the consumer more and more. In this competitive scenario, gamification represents a digital marketing tool that strongly differentiates a company and creates value for its customers. To further differentiate and create a unique experience, luxury companies may consider developing and integrating a game app with the e-commerce website, to the point that the e-commerce website could evolve into a luxury gaming experience. This experience would take into account the current preferences of the new generation of luxury consumers, specifically Millennials and Generation Z. The evolution of the e-commerce website towards an engaging luxury gaming experience may include not only playful and personalization elements but new values that new generations are seeking in luxuries, such as inclusion and the enhancement of diversity. This latter issue, concerning the inclusion of in-game mechanisms of relevant values for the new generations, represents a possible avenue for future research.

Finally, this paper shows some limitations that can be the basis for further studies. An evident limitation of our study is that it builds on a single case study of an online multi-brand retailer in the luxury context. That means the external validity of our findings in other contexts, such as the non-luxury or non-retailing ones, needs to be verified. Furthermore, the development and adoption of gamification techniques and the features may be different beyond the boundaries of luxury and retailing. Additionally, the paper considered only one emblematic case of an innovative player in multi-brand retailing that succeeded in launching a game app. A multiple-case study approach might be helpful to build a comparison and allow generalization. Moreover, future research could study the role of gamification for analytics purposes. For example, it could be interesting to understand the player’s behavior in the game in terms of products selection and different combinations. Hence, how gamification can enhance the understanding of consumers’ digital behavior represents a valuable avenue for future research. Finally, this paper addressed the topic of gamification from an online multi-brand luxury retailer's perspective by highlighting the benefits and, more in general, the positive side of gamification as an effective digital marketing tool. However, future research should reverse this perspective by investigating gamification's 'dark side' regarding risks and difficulties for business purposes.

## Footnotes

1 https://uk.fashionnetwork.com/news/Luxury-industry-comes-to-terms-with-gen-z-s-appetite-for-collaborations-resale,1091093.html.
